# Supplementary material for: Structure of a mitochondrial ATP synthase with bound native cardiolipin
Source: eLife. 2019 Nov 18;8:e51179. doi: 10.7554/eLife.51179 (PMC6930080; doi:10.7554/eLife.51179)
Supplement: Supplementary file 1. [file elife-51179-supp1.docx]

Table S1: Cryo-EM data collection

| Electron Microscope | Titan Krios |
| --- | --- |
| Camera | K2 Summit (counting mode) |
| Voltage | 300 kV |
| Energy filter slit width | 20 eV |
| Nominal Magnification | 130,000 x |
| Calibrated pixel size | 1.05 Å |
| Total exposure | 36.3 electrons/Å^2^ |
| Exposure rate | 4 electrons/(pixel · s) |
| Number of frames | 20 |
| Defocus range | −1.6 to −3.6 μm |
