# Supplementary material for: Structure of a mitochondrial ATP synthase with bound native cardiolipin
Source: eLife. 2019 Nov 18;8:e51179. doi: 10.7554/eLife.51179 (PMC6930080; doi:10.7554/eLife.51179)
Supplement: Supplementary file 2. [file elife-51179-supp2.docx]

**Supplementary File 2:** *E. gracilis* ATP synthase dimer atomic model statistics

*FSC corrected for the effect of the mask according to 0.143-cutoff criterion

| **atomic model** | **membrane region** | **F_1_/c-ring class-1** | **F_1_/c-ring class-2** | **F_1_/c-ring class-3** | **peripheral stalk**  **class-1** | **rotor**  **class-1** | **composite**  **dimer** |
| --- | --- | --- | --- | --- | --- | --- | --- |
| map resolution (Å)* | 2.82 | 3.04 | 3.14 | 3.92 | 3.82 | 3.30 | 4.32 |
| CC (map/model) | 0.82 | 0.7 | 0.81 | 0.81 | 0.78 | 0.78 | 0.74 |
| resolution (map/model)** | 2.98 | 3.43 | 3.20 | 3.85 | 3.95 | 3.4 | 4.02 |
| PDB accession code | 6TDV | 6TDY | 6TDZ | 6TE0 | 6TDW | 6TDX | 6TDU |
| EMDB accession code | EMD-10468 | EMD-10471 | EMD-10472 | EMD-10473 | EMD-10469 | EMD-10470 | EMD-10467 |
| model composition |  |  |  |  |  |  |  |
| # atoms | 95422 | 81812 | 81735 | 79283 | 13006 | 20303 | 271333 |
| # protein residues | 5464 | 5289 | 5283 | 5136 | 841 | 1339 | 16878 |
| # lipids | 37 | - | - | - | - | - | 37 |
| # ATP/ADP | - | 4/1 | 4/1 | 4/1 | - | - | 8/2 |
| # Mg ions | - | 5 | 5 | 5 | - | - | 10 |
| B-factor (Å^2^) |  |  |  |  |  |  |  |
| - protein (mean) | 49.39 | 6.72 | 64.20 | 85.96 | 67.71 | 49.99 | 161.27 |
| -ligand (mean) | 44.70 | 38.33 | 38.03 | 44.77 | - | - | 88.02 |
| rotamer outliers (%) | 0 | 0 | 0 | 0 | 0 | 0 | 0.01 |
| Ramanchandran (%) |  |  |  |  |  |  |  |
| - outliers | 0 | 0.11 | 0 | 0.02 | 0 | 0 | 0.10 |
| - allowed | 2.62 | 4.00 | 3.60 | 4.03 | 7.31 | 2.52 | 96.55 |
| - favored | 97.38 | 95.89 | 96.40 | 95.95 | 92.69 | 97.48 | 3.35 |
| clash score | 0.96 | 2.71 | 2.54 | 2.72 | 1.77 | 2.22 | 1.43 |
| MolProbity score | 0.91 | 1.33 | 1.28 | 1.34 | 1.39 | 1.10 | 1.10 |
| RMSD |  |  |  |  |  |  |  |
| - bonds (Å) | 0.005 | 0.007 | 0.008 | 0.005 | 0.005 | 0.008 | 0.003 |
| - angles (°) | 0.728 | 0.796 | 0.756 | 0.746 | 0.721 | 0.826 | 0.755 |
| EMRinger score | 4.43 | 1.14 | 3.44 | 2.25 | 1.44 | 2.79 | 1.16 |

** FSC (masked) according to 0.5-cutoff criterion
