# Supplementary material for: Structure of a mitochondrial ATP synthase with bound native cardiolipin
Source: eLife. 2019 Nov 18;8:e51179. doi: 10.7554/eLife.51179 (PMC6930080; doi:10.7554/eLife.51179)
Supplement: Supplementary file 3. [file elife-51179-supp3.docx]

| ***E.gracilis* subunits** | ***E.gracilis* subunit identifier** | **GenBank  accession code** | **ORF MW  [kDa]** | **previously identified in *E. gracilis*** | ***S. cerevisiae* gene nomenclature/ name in previous identification** | **comments** | ***Euglena-Trypanosoma*  BLAST E-value** |
| --- | --- | --- | --- | --- | --- | --- | --- |
| **α** | comp27580_c0_seq1 | GDJR01036292.1 | 61,84 | universally conserved | ATP1 | canonical F_1_ | - |
| **β** | comp30487_c1_seq1 | GDJR01048402.1 | 53,17 | universally conserved | ATP2 | canonical F_1_ | - |
| **γ** | EG_transcript_19235 | GEFR01019235.1 | 35,07 | universally conserved | ATP3 | canonical F_1_ | - |
| **δ** | comp8147_c0_seq1 | GDJR01108837.1 | 19,54 | universally conserved | ATP16 | canonical F_1_ | - |
| **ε** | comp36626_c0_seq1 | GDJR01100113.1 | 8,74 | universally conserved | ATP15 | canonical F_1_ | - |
| **p18** | comp36594_c0_seq1 | GDJR01100103.1 | 20,97 | Yadav et al, Perez et al | p18 | euglenozoa-specific F_1_ | - |
| **IF_1_** | EG_transcript_49190 | GEFR01049155.1 | 11.67 | universally conserved | INH1 | F_1_ inhibitor | - |
| **OSCP** | comp36635_c0_seq1 | GDJR01100117.1 | 29.53 | universally conserved | ATP5 | F_o_ yeast homolog | - |
| **subunit *a*** | sga_contig_684056 | - | 32.61 | newly identified | ATP6 | F_o_ yeast homolog | - |
| **subunit *b*** | comp8208_c0_seq1 | GDJR01108903.1 | 12.66 | newly identified | ATP4 | F_o_ yeast homolog | - |
| **subunit c** | EG_transcript_45706 | GEFR01045692.1 | 10.81 | universally conserved | ATP9 | F_o_ yeast homolog | - |
| **subunit *d*** | comp20520_c0_seq1 | GDJR01017994.1 | 53.87 | Yadav et al, Perez et al | ATP7/ATPTB2 | F_o_ yeast homolog | 0.19 |
| **subunit *f*** | VDSF_6_rev_Run01_Cp357_MD1/ G11.esd | EC611052.1 | 11.18 | newly identified | ATP17 | F_o_ yeast homolog | - |
| **subunit *i/j*** | comp12674_c0_seq1 | GDJR01005239.1 | 12.51 | Yadav et al | ATP18/ATPTB10/ UP19 | F_o_ yeast homolog | 0.063 |
| **subunit *k*** | EG_transcript_55785 | GEFR01055688.1 | 12.73 | Yadav et al | ATP19/UP21 | F_o_ yeast homolog | - |
| **subunit 8** | sga_contig_684056 | KT732265.1 | 7.01 | newly identified | ATP8 | F_o_ yeast homolog | - |
| **ATPTB1** | EG_transcript_7573 | GEFR01007572.1 | 55.93 | Yadav et al, Perez et al | ATPTB1 | F_o_ *Trypanosoma* homolog | 2E-6 |
| **ATPTB3** | EG_transcript_14428 | GEFR01014423.1 | 35.14 | Yadav et al, Perez et al | ATPTB3 | F_o_ *Trypanosoma* homolog/putative isocitrate dehydrogenase homolog | 8E-5 |
| **ATPTB4** | comp8167_c0_seq1 | GDJR01108860.1 | 18.81 | Yadav et al, Perez et al | ATPTB4 | F_o_ *Trypanosoma* homolog | 9E-24 |
| **ATPTB6** | EG_transcript_28774 | GEFR01028782.1 | 21.71 | Yadav et al, Perez et al | ATPTB6 | F_o_ *Trypanosoma* homolog | 7E-11 |
| **ATPTB12** | comp12685_c0_seq1 | GDJR01005257.1 | 11.35 | Yadav et al, Perez et al | ATPTB12 | F_o_ *Trypanosoma* homolog | 9E-8 |
| **ATPEG1** | comp12813_c0_seq1 | GDJR01005511.1 | 19.72 | Yadav et al | UP14 (comp51661_c0_seq2) | no homolog outside *Euglena* identified | - |
| **ATPEG2** | EG_transcript_33701 | GEFR01033662.1 | 16.18 | Yadav et al | UP15comp46683_c0_seq1_cut | no homolog outside *Euglena* identified | - |
| **ATPEG3** | comp17420_c0_seq1 | GDJR01012366.1 | 13.79 | Yadav et al | UP18(gi \| 109777597_cut) | no homolog outside *Euglena* identified | 4E-6 |
| **ATPEG4** | EG_transcript_49361 | GEFR01049309.1 | 13.69 | Yadav et al | UP16 (comp48845_c0_seq1_cut) | no homolog outside *Euglena* identified | - |
| **ATPEG5** | EG_transcript_70983 | GEFR01071033.1 | 10.81 | newly identified | - | cytochrome c oxidase assembly factor 6, putative homolog | - |
| **ATPEG6** | comp30797_c0_seq1 | GDJR01049874.1 | 9.22 | newly identified | - | no homolog outside *Euglena* identified | - |
| **ATPEG7** | comp8129_c0_seq1 | GDJR01108818.1 | 8.85 | newly identified | - | no homolog outside *Euglena* identified | - |
| **ATPEG8** | comp8203_c0_seq1 | GDJR01108900.1 | 7.43 | newly identified | - | no homolog outside *Euglena* identified | - |

**Table S3: Subunits of the *E. gracilis* ATP synthase dimer identified in this study.**
